# Genome-wide association study to identify potential genetic modifiers in a canine model for Duchenne muscular dystrophy

**DOI:** 10.1186/s12864-016-2948-z

**Published:** 2016-08-22

**Authors:** Candice Brinkmeyer-Langford, Cynthia Balog-Alvarez, James J. Cai, Brian W. Davis, Joe N. Kornegay

**Affiliations:** 1 Department of Veterinary Integrative Biosciences, Texas A&M University, College Station, Texas, USA; 2 Cancer Genetics Branch, National Human Genome Research Institute, National Institutes of Health, Bethesda, MD USA

**Keywords:** Muscular dystrophy, Duchenne muscular dystrophy, DMD, Golden retriever muscular dystrophy, GRMD, Modifier, Linear mixed-model analysis, Gene expression

## Abstract

**Background:**

Duchenne muscular dystrophy (DMD) causes progressive muscle degeneration, cardiomyopathy and respiratory failure in approximately 1/5,000 boys. Golden Retriever muscular dystrophy (GRMD) resembles DMD both clinically and pathologically. Like DMD, GRMD exhibits remarkable phenotypic variation among affected dogs, suggesting the influence of modifiers. Understanding the role(s) of genetic modifiers of GRMD may identify genes and pathways that also modify phenotypes in DMD and reveal novel therapies. Therefore, our objective in this study was to identify genetic modifiers that affect discrete GRMD phenotypes.

**Results:**

We performed a linear mixed-model (LMM) analysis using 16 variably-affected dogs from our GRMD colony (8 dystrophic, 8 non-dystrophic). All of these dogs were either full or half-siblings, and phenotyped for 19 objective, quantitative biomarkers at ages 6 and 12 months. Each biomarker was individually assessed. Gene expression profiles of 59 possible candidate genes were generated for two muscle types: the cranial tibialis and medial head of the gastrocnemius. SNPs significantly associated with GRMD biomarkers were identified on multiple chromosomes (including the X chromosome). Gene expression levels for candidate genes located near these SNPs correlated with biomarker values, suggesting possible roles as GRMD modifiers.

**Conclusions:**

The results of this study enhance our understanding of GRMD pathology and represent a first step toward the characterization of GRMD modifiers that may be relevant to DMD pathology. Such modifiers are likely to be useful for DMD treatment development based on their relationships to GRMD phenotypes.

**Electronic supplementary material:**

The online version of this article (doi:10.1186/s12864-016-2948-z) contains supplementary material, which is available to authorized users.

## Background

Duchenne muscular dystrophy (DMD) is an X-linked disease that causes progressive muscle degeneration in approximately 1 out of 5,000 boys [1]. Skeletal and cardiac muscle can both be affected, and, while the disease is lethal, clinical presentations of DMD vary from patient to patient (e.g. age at diagnosis, age at loss of ambulation, body mass index, and lifespan [2, 3]). DMD patients are often wheelchair-bound by age 13 [4] and typically succumb to cardiomyopathy and/or respiratory complications before age 30. The primary molecular cause of DMD is the absence of functional dystrophin, a protein required for proper muscle function. Mutations in the dystrophin gene, *DMD* [5], have been catalogued extensively in humans (e.g. [2, 6–8]) and it is apparent that the level of functional dystrophin is a key factor in determining the severity of disease. In-frame mutations resulting in truncated protein products cause the less-severe Becker muscular dystrophy, while mutations disrupting the reading frame cause the more-severe DMD [9]. However, this “reading-frame rule” does not always apply: cases have been documented of children without any detectable dystrophin production who display phenotypes so mild that clinical diagnosis is ambiguous [10, 11].

Canine models for human diseases offer perspectives not provided by smaller animal models. Dogs are more physically comparable in size to humans than are mice; canine orthologs are available for approximately 92 % of human genes [12]. The breeding structure used to propagate many dog breeds supports breed-specific stretches of genetic homogeneity, making the task of identifying contributing genes more facile in dogs as causal alleles segregate on a relatively homogeneous background. Colonies of dogs have been invaluable for understanding human conditions and developing treatments (for example, Alport syndrome [13]; vaccine response [14]); careful record-keeping and cooperation by dog owners, veterinarians and researchers can also benefit disease research [15].

Dog models for DMD have phenotypes that are very similar to the human condition, making them powerful for developing and testing new treatments. Preclinical studies from dogs can predict the potential success of a treatment in humans, as dog models of DMD are genetically and phenotypically comparable to the human disease [16] and, compared to mice, dogs are similar to humans in body and organ size and immune response [17]. Golden retriever muscular dystrophy (GRMD) is the most extensively studied of the canine models of dystrophin-deficient muscular dystrophy. GRMD is remarkably similar to DMD both clinically and pathologically [18, 19]. Like DMD, dogs with GRMD are afflicted with a progressive, fatal disease with limb skeletal muscle, cardiac and respiratory involvement [20].

While DMD can be caused by many different types of mutations in humans, GRMD is caused by a single splice-site mutation that results in a frame shift and prematurely truncated, nonfunctional protein product [21]. GRMD carrier females can be artificially inseminated by GRMD-affected males to produce GRMD-affected females, which do not differ phenotypically from males; affected females (aside from a few rare “manifesting carriers” [22, 23]) have not been seen in humans.

As a first step toward characterizing genetic modifiers of GRMD, we performed a pilot study using linear mixed-model (LMM) analysis of 8 variably-affected dystrophic dogs and 8 non-dystrophic dogs, all either full or half-siblings, with complete data available for age-specific phenotypic measurements. We have also compared our LMM findings with a cohort of unrelated dogs to evaluate the GRMD population relative to a broader population. GRMD disease grows progressively worse with age and marked variation in disease severity is observed between different muscles [20, 24], which suggests that genetic modifiers of GRMD disease act differentially between muscles. We have therefore included two different age groups and two muscle types (the cranial tibialis – a flexor, and the medial head of the gastrocnemius – an extensor) in our analysis to evaluate how gene expression changes with each variable. These dogs were extensively phenotyped using biomarkers that correlate with disease severity on multiple levels (for example, force generated by different muscle groups).

These LMM analyses provide a foundation for future research, including fine-mapping of phenotype-associated modifier loci, targeted sequencing, and proteomics.

## Methods

### Animals

All GRMD dogs used in this study were from the colony at the University of North Carolina at Chapel Hill, now located at Texas A&M University. The dogs were maintained and treated according to the standards of the National Research Council Guide for the Care and Use of Laboratory Animals. Blood creatine kinase levels taken shortly after birth were used to diagnose neonates with GRMD [19], along with PCR as previously described [25]. The colony is primarily maintained by mating hemizygous GRMD males to carrier females to produce approximately equal numbers of affected males and females.

Sixteen dogs were selected for this study based on the availability of phenotypic data. This cohort included 8 GRMD-affected and 8 unaffected dogs from two age groups: ~6 months (*n* = 9) and ~12 months (*n* = 7) of age. The 6-month-old group contained 4 normal (all male) and 5 affected (1 female and 4 males); the 12-month-old group contained 4 normal (all male) and 3 affected (2 females and 1 male). These 16 dogs represented 5 litters; 13 of the dogs were half-sibs and the remaining 3 were cousins to the other dogs. Three of the 5 litters included both normal and affected dogs.

**Fig. 1:**
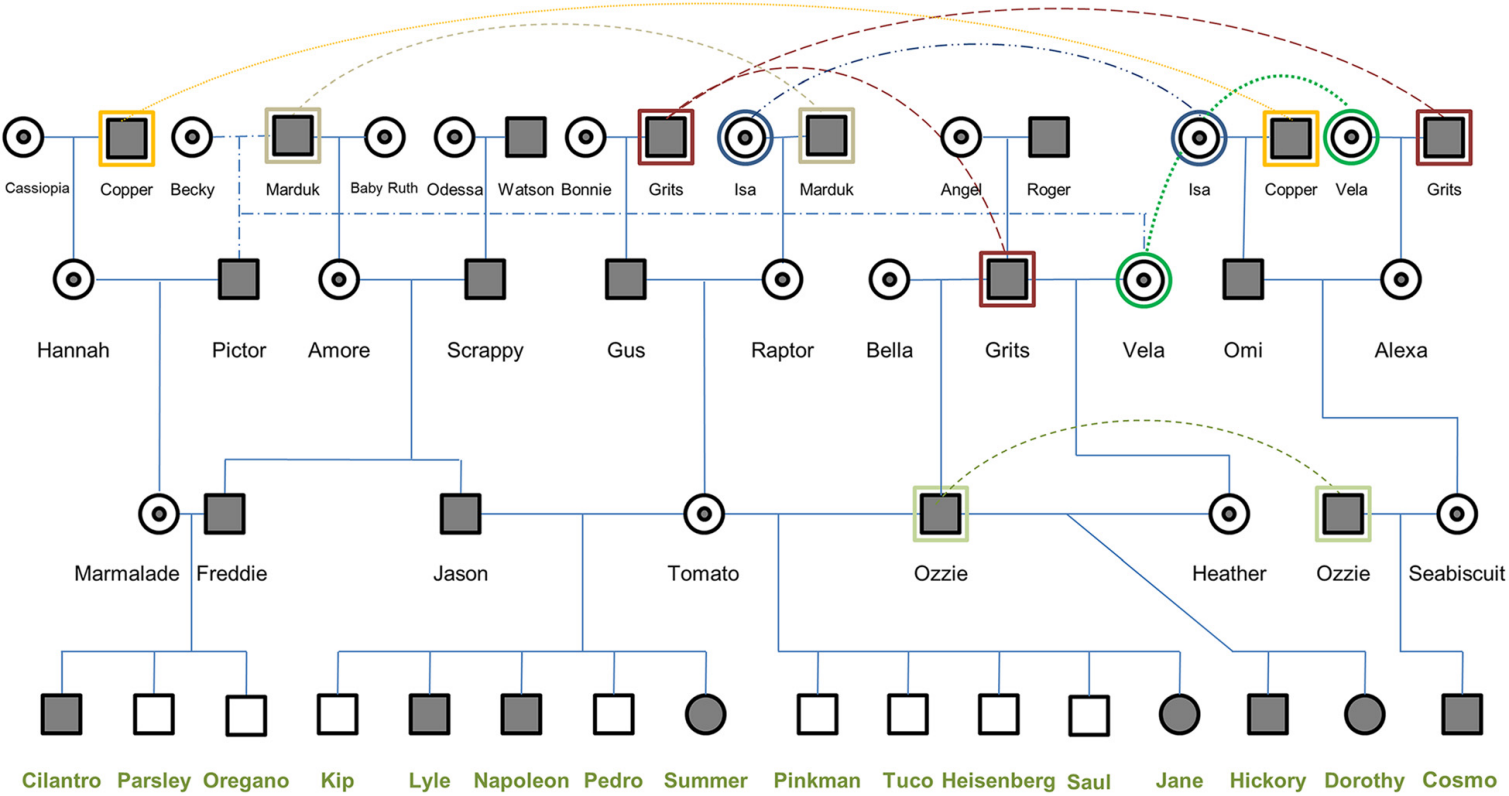
Pedigree representing the ancestry of the 16 dogs used in this study. Circles represent females; squares represent males; open symbols represent non-GRMD (non-dystrophic); blackened symbols represent GRMD (dystrophic); a small circle within a larger circle represents GRMD carrier females; animals listed in more than one position within the pedigree are indicated by a larger circle or square encompassing the primary symbol for the animal, along with a dotted line connecting the multiple positions for that animal in the pedigree. The 16 dogs investigated for this study are listed across the bottom of the pedigree, with names printed in bold green font

Also included in our study were the 7 unique parents to the 16 phenotyped dogs. These parents were not phenotyped, but were included to allow us to evaluate linkage. A pedigree of the dogs is presented in Fig. 1.

Genotype data was also obtained for 19 unrelated and unaffected golden retrievers for which muscle tissue samples were not available.

### Necropsies

Samples of muscle tissue were collected from the medial head of the gastrocnemius and cranial tibialis at necropsy, flash-frozen in liquid nitrogen, and subsequently stored at −80 °C.

### Biomarker measurements

The phenotypes/biomarkers evaluated in this study were developed for use in preclinical trials in GRMD dogs, and are objective and quantitative. Detailed descriptions of each biomarker and the method(s) of measurement have been published previously [20, 24, 26–32] and include: body-mass-corrected tibiotarsal joint (TTJ) tetanic flexion and extension force (Newtons/kg; [24]), tibiotarsal joint angle (degrees; [28]), eccentric contraction decrement (ECD) at 10 and 30 stimulations (percent; [29, 30]), maximum hip flexion angle (degrees; [28]), pelvic angle (degrees; [28]), body-mass-corrected cranial sartorius circumference (mm/kg; [20]), quadriceps femoris weight (g) and quadriceps femoris weight corrected for body weight (g/kg) [20], body weight (in kg) measurements taken at 1 month, 6 months, and 12 months of age [24], and ratios of body weight to birth weight for each time point (percent) [24].

The biomarkers used for this study provide objective indices of disease severity. Because pathologic data were collected at necropsy, we were able to assess terminal muscle weights to gain further insight on the degree of atrophy or hypertrophy. As an example, absolute and body-mass-corrected weight of the quadriceps femoris, the principal muscle involved in stifle (knee) extension was assessed. Other biomarkers assessed functional parameters relevant to specific muscles used for validation of candidate genes. Most notably, the cranial tibialis and gastrocnemius are the principal muscles contributing to TTJ tetanic flexion and extension, respectively. Tibiotarsal joint, maximum hip flexion, and pelvic joint angle measurements provided objective insight on the severity of debilitating contractures and postural instability in GRMD and served as surrogates for overall muscle imbalance and weakness that ultimately cause loss of ambulation in severely affected dogs [33].

### DNA extraction

DNA was extracted from tissue sections using the phenol-chloroform-isoamyl alcohol method [34], as modified by D. Ish-Horowitz [35].

### Genotyping

DNA samples from the 16 GRMD affected and control dogs, along with the 7 parental samples, were genotyped on the Illumina CanineHD BeadChip (Illumina Inc, San Diego, CA) and scored at GeneSeek (Lincoln, NE). Furthermore, DNAs from 19 golden retrievers, unrelated to the colony as well as to one another, were genotyped on the same array to increase confidence in our results and to evaluate the GRMD population relative to a broader population, as described below.

### Association studies

Linear mixed-model (LMM) analyses were performed using three different methods to reduce the number of false associations identified, and to overcome biases inherent to each methodology. Data from all dogs were assessed by all three algorithms, which perform linear mixed models based on clustering to account for both population substructure and relatedness via a kinship matrix estimated from identical by descent distances.

Associations between SNPs and biomarkers were initially identified using the whole genome association analysis program plink [36]. Each biomarker was assessed separately as its own endophenotype. SNPs were filtered and pruned to reduce spurious associations. Inclusion thresholds were 10 % maximum per-SNP missing, 5 % maximum per-dog missing, and 10 % minimum minor allele frequency (MAF). After applying these filters, 125,665 SNPs remained for analysis on all 16 dogs. Population stratification was estimated for each endophenotype using the genomic control λ value calculated by plink; a λ value of 1 indicates no stratification, meaning association results are not likely to be influenced by population structure [37]. For plink results, adjustment for multiple testing was done by using Bonferroni procedure with *p*-value cutoff of 3.98 × 10^−7^ (=0.05/125665) to give *p*_*genome-wide*_ < 0.05.) We subsequently performed association studies using Genome-wide efficient mixed-model association (GEMMA) [38] and SNP and Variation Suite (SVS) (version 8.1.1) software to confirm the robustness of associations, similar to strategies used in previous studies [39–41]. The linear mixed-model analysis GEMMA allowed us to eliminate false associations due to breeding structure. We performed three association tests: the Wald test, likelihood ratio test, and score test, as described in the GEMMA manual [42]. We furthermore used the Efficient Mixed-Model Association (EMMA) approach in SVS with the EMMAX (EMMA eXpedited) technique for normalizing the kinship matrix [43, 44]. Results were further analyzed using R software [45] to develop manhattan plots to visualize associations. Because the GRMD population used in this study consists of related individuals, we used multidimensional scaling (MDS) via plink to identify sample clustering and population stratification with and without the unrelated population of 19 golden retrievers.

The causal mutation responsible for GRMD has been previously identified in the *DMD* gene [21] (located on the canine X chromosome, positioned between bases 26290903–28444623; canine genome assembly version 3.1, Sept. 2011). Association with SNPs residing near or within the *DMD*, using case–control analyses, confirmed the causal relationship between GRMD and the *DMD* gene.

### SNP effect prediction

All SNPs found to be statistically-significant via LMM analysis were evaluated to determine possible effects of SNPs on genes, transcripts, and protein sequences using Ensembl’s Variant Effect Predictor [46].

### Haplotyping and linkage disequilibrium

Since contributing loci are likely to be co-inherited with significantly-associated SNPs, we developed haplotypes using plink software [36] for those chromosomes containing significantly associated SNPs. Blocks of linkage disequilibrium (LD) were then visualized with Haploview [47].

### Candidate modifier gene identification

Chromosomes and regions of LD found to harbor SNPs statistically associated with one or more biomarkers were manually examined to identify candidate genes. We used the BioMart tool from the Ensembl genome browser to extract information about positions and functions of genes within regions of interest in the canine genome, together with information about human orthologs, where available. At the same time, we searched the NCBI database for gene information, including associated phenotypes. Both databases provided information about gene ontologies and interactions, providing additional evidence to support possible roles for candidate genes modifying GRMD phenotypes. Relevant publications, listed in the Bibliography section of the gene reference pages on NCBI, and Gene References into Function (GeneRIFs) were also used to identify potential candidate genes. Genes were selected for qPCR analysis if their known or predicted function(s) were consistent with the pathogenesis of muscular dystrophy (such as roles in skeletal muscle or cardiac function), or if they interacted with other gene(s) or pathway(s) relevant to the GRMD phenotypes being studied.

### RNA extraction

Total RNA was extracted from necropsy samples from the cranial tibialis and medial head of the gastrocnemius muscles archived at −80 °C for each of the 16 dogs using TriPure Isolation Reagent (Roche; Indianapolis, IN) as per manufacturer’s instructions. RNA was precipitated using isopropanol and 75 % EtOH, and dissolved in ultra-pure water. To minimize DNA contamination, samples were DNase-treated using the DNA-Free DNase Treatment and Removal kit (Ambion). The RNA concentrations in the individual samples were measured using a Nanodrop 2000 spectrophotometer and assessed for quality on a 2100 Bioanalyzer (Agilent Technologies, Santa Clara, CA). The RIN values ranged from 7.5 – 8.2, with the exception of one sample which had a RIN value of 6.7. The Standards read 9.

### Quantitative PCR (qPCR)

Total RNA was directly reverse transcribed to cDNA using SuperScript II (Invitrogen). Briefly, samples of skeletal muscle DNase-treated RNA (100 ng) were reverse transcribed with oligo-dT and random primers and Superscript II (Invitrogen, Carlsbad, CA). The reverse transcription reactions consisted of 100 ng of DNase-treated RNA in a 50 μl reaction, ultra pure water, oligo dT (2.5 μl of 500 ng/μl) and random hexamer (0.48 μl of 1 mM stock) heated to 65 °C for 5 min and cooled to room temperature. Next, Superscript II (2 μl), 5X 1st Strand buffer (10 μl), 0.1 M DTT (5 μl), 10 mM dNTPs (2.5 μl) and an RNase block Ambion’s Superasin (1 μl) were added to the mix. All were heated to 37 °C for one hour followed by reverse transcription inactivation at 90 °C for 5 min.

TaqMan® Gene Expression assays (Life Technologies, Grand Island, NY; [48, 49]) were used to measure expression levels for 51 genes identified within candidate gene regions. qPCR using TaqMan assays was performed using MicroAmp Fast Optical 96 well plates (Applied Biosystems). The qPCR reactions for each TaqMan assay consisted of 10 μl TaqMan Gene Expression Master Mix, 1 μl TaqMan assay, 8.5 μl PCR grade water, and 0.5 μl of each reverse transcription reaction (cDNA) for a total of 20 μl per well. TaqMan assays were not available for an additional 8 genes; for these, we used SYBR Green technology to determine expression levels. Primers for genes assessed via SYBR Green were designed from two neighboring exons flanking one intron (when possible), or from a single exon, using Primer3 software (http://biotools.umassmed.edu/bioapps/primer3_www.cgi; [50]). Each 20 μl reaction contained 10 μl Power SYBR Green PCR Master Mix (Applied Biosystems), 2 μl each forward and reverse primers (3 μM each), 5.5 μl ultra-pure water, and 0.5 μl (1 ng) cDNA for a total of 20 μl per well. All qPCR reactions were performed in duplicate using the 7900HT Fast qPCR System (Applied Biosystems); primer and assay information is listed in Additional file 1: Table S1. The cycling parameters on the 7900HT machine were 50 °C for 2 min, 95 °C for 10 min, and cycling 40 repeats of 95 °C for 15 s and 60 °C for 1 min. The parameters were the same for the Sybr assays with a dissociation curve added to validate primers.

No single housekeeping gene could be identified as having steady expression levels across both muscle types and age groups interrogated; therefore we used the geometric mean strategy for data normalization, in which the normalization factor is calculated using multiple genes, as described [51]. Differential gene expression was statistically evaluated via unpaired *t*-test using GraphPad QuickCalcs Web site: http://graphpad.com/quickcalcs/ttest1.cfm (accessed July 2014 [52]). Expression levels were considered significant at *p* < 0.05.

### Linear regression correlating gene expression with clinical phenotype measurements

Simple linear regression analyses between specific GRMD quantitative phenotypes and expression values obtained for each GRMD-affected dog were performed for each of the 39 potential candidate genes identified by the LMM analyses. All phenotype-transcript regression pairs with a correlation coefficient (*R*^2^) value of ≥0.8 were considered significant.

## Results

### Association studies

#### Genomic control factors

Case/control association using plink confirmed the causal mutation on *Canis familiaris* chromosome X CFAX, with a genomic inflation factor λ = 1.

LMM analyses for other biomarkers in GRMD dogs, performed using plink, resulted in different genomic inflation factors (λ) (Table 1). λ values larger than 1.05 indicate confounders such as population-level stratification or family structure [39]; in this study, strong familial structure is the likely culprit for some of the high λ values. However, several biomarkers (shaded in Table 1) were found to be relatively robust against stratification. These biomarkers, therefore, appear to be the most reliable indicators of GRMD disease severity independent of family structure.

**Table 1:** Genomic inflation factors calculated for GRMD biomarkers. The biomarkers that are the most robust against stratification are shaded

λ	Biomarker
1.26	TTJ Tetanic flexion (N/kg)
1	TTJ Tetanic extension (N/kg)
1	TJA (degrees)
1.01	Percent eccentric contraction decrement (at 10 stimulations)
1	Percent eccentric contraction decrement (at 30 stimulations)
1	Maximum hip flexion angle
1	Pelvic angle
1.14	Cranial sartorius circumference (mm/kg)
1.23	Quadriceps femoris weight (g)
1.05	Quadriceps femoris weight (g/kg body weight)
1.38	Body weight at birth (kg)
1.53	Body weight at age 1 month (kg)
1.99	Percent body weight gain: birth-1 month
1	Body weight at age 6 months (kg)
1	Percent body weight gain: birth-6 months
1.22	Body weight (kg) at age 12 months
1.5	Percent body weight gain: birth-12 months

#### Population stratification

**Fig. 2:**
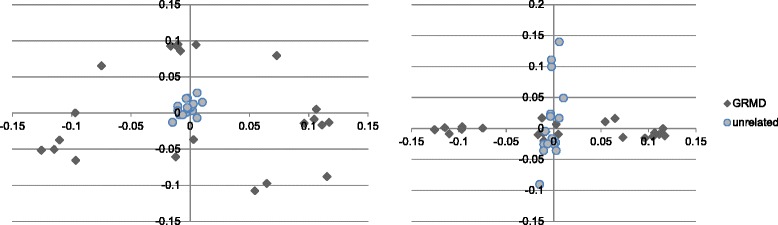
Multi-dimensional scaling (MDS) plots showing the genomic kinship between GRMD and unrelated golden retriever dogs, based on a set of 125,665 SNPs (filtered based on inclusion parameters as described in Methods section). These graphs illustrate the distributions of two different sets of coordinates across both populations. The unrelated dogs tended to cluster together near the center of the plots, while GRMD dogs appeared as genetically more distant from each other

MDS plots were constructed to visualize population stratification, which could influence the relevance of variants identified as significant. No stratification was observed in the GRMD and parental samples. When the 19 unrelated golden retriever samples were added to the total population and the results plotted, the unrelated samples clustered tightly together, with GRMD samples dispersed around on all sides of the unrelated sample cluster (Fig. 2). This distribution reflects the nature of the breeding strategy used for this GRMD colony, and the resulting genetic diversity, which reduces population stratification. Unrelated animals are added to the breeding stock once the cumulative inbreeding coefficient of the colony reaches ~0.20 (the point at which neonatal mortality reaches 22 %) [31]. As a result, the GRMD colony possesses many of the advantages of an inbred population (e.g. reduced influence of confounding factors such as environmental variation), while capturing genetic diversity more similar to that found in human populations. In summary, the significant associations discovered using the GRMD population are likely applicable to other populations as well.

#### Significant associations

We found 99 SNPs to be significantly associated with GRMD phenotypic variation (FDR *p* <0.05). Following Bonferroni correction, 7 of these SNPs also met significance criteria (*p*_*Bonferroni*_ < 0.05) and 2 more showed a suggestive association (*p*_*Bonferroni*_ < 0.1). These 9 SNPs were located on 4 chromosomes in regions near or within genes with functions relevant to GRMD phenotype, including CFA7, 9, 23, and X (listed in Table 2; base pair [bp] positions given are from assembly version 3.1 of the canine genome, known as CanFam3.1). Findings from analyses using GEMMA and SVS concurred with the plink results, adding credibility to the robustness of SNP associations found with plink. Results from all SNP association experiments are available in Additional file 2: Table S2.

**Table 2:** Nine SNPs were most strongly associated with GRMD biomarkers following Bonferroni correction. The first column lists canine chromosome number and second column lists base-pair location of the SNP (based on CanFam3.1). The third column gives the SNP identifier

Chromosome	bp position	SNP identity
7	65462481	BICF2P767388
7	74444198	TIGRP2P104104_rs8716172
9	51783485	TIGRP2P130620_rs8982512
9	52461811	TIGRP2P130855_rs9077374
23	12784487	BICF2P1005915
X	26396900	BICF2P760963
X	27322875	BICF2G630533008
X	28076757	BICF2S2291136
X	25639583	BICF2G630533758

### SNP effects

SNPs from the associated regions on CFA7, 9, 23, and X were evaluated for potential consequences using the Ensembl Variant Effect Predictor tool [46]. No SNPs were found to affect amino acid sequences, or alter codons. Furthermore, no co-located variations were found. The Variant Effect Predictor output is provided in Additional file 3: Table S3.

### Haplotypes

**Fig. 3:**
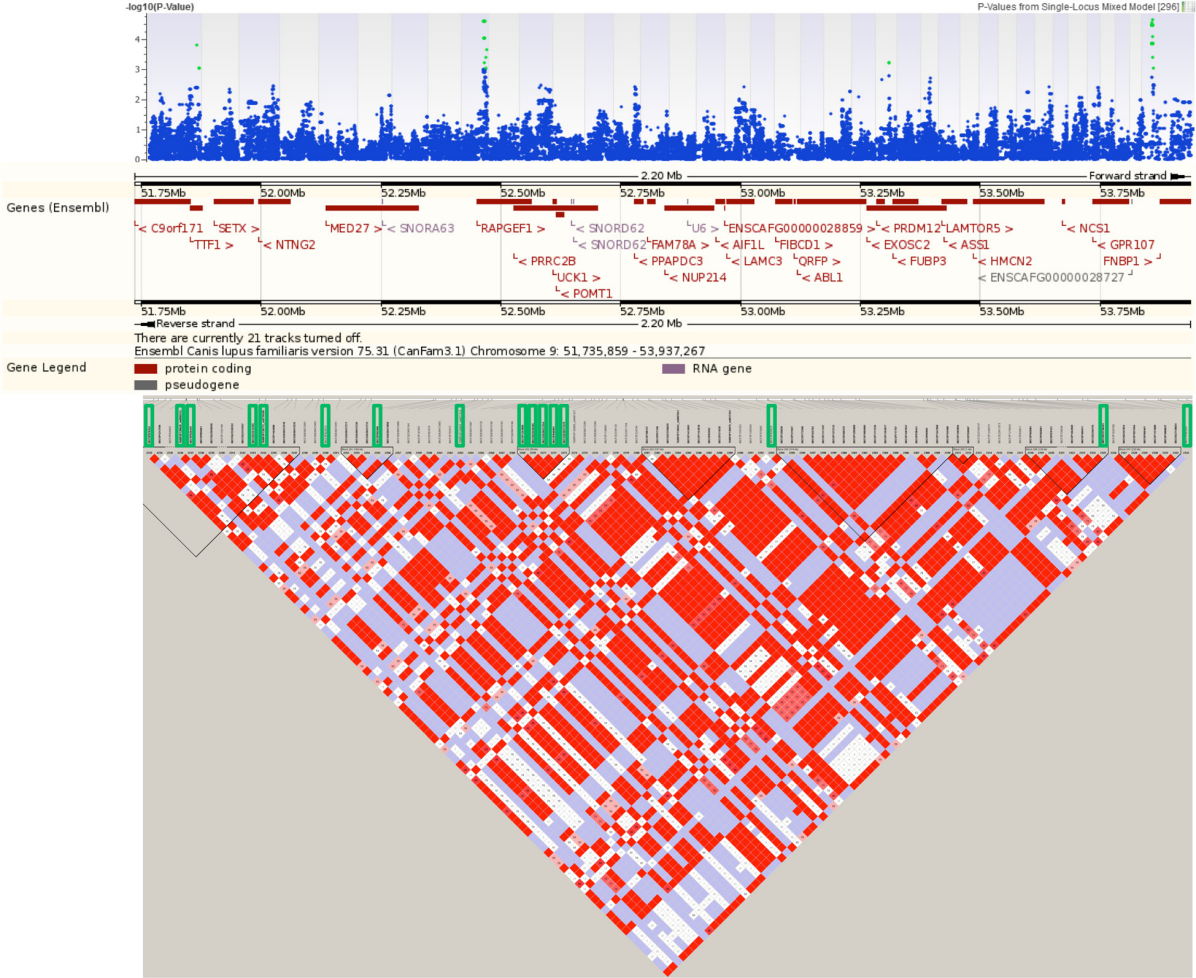
Association between tetanic flexion and loci on CFA9. In panel A, a Manhattan plot spotlights 16 significant SNPs (*p* < 0.01) associated with tetanic flexion (in green), which suggest a relationship between this biomarker and one or more loci on CFA9. These SNPs are clustered between bases 51735859–53937266 on CFA9; this region is illustrated in panel B. Panel C shows LD within this specific region in the GRMD dogs evaluated in this study. The 16 significant SNPs are again spotlighted here, with green rectangles around the SNP names. Gene *RAPGEF1* is located within a region that is in LD with several of these significant SNPs; qPCR results confirmed that the expression of this gene in the cranial tibialis is associated with tetanic flexion

We evaluated LD and identified haplotype blocks for CFA7, 9, 23, and X. Closely-located candidate modifier genes within regions of strong LD may be inherited with significant SNPs; therefore, we initially focused our gene search on the regions surrounding significant SNPs. For example, see Fig. 3, which shows a region on CFA9 that contains SNPs in LD with each other and with SNPs within the *RAPGEF1* gene. However, due to the relatively outbred nature of the GRMD colony and resulting lack of LD, we did not limit our focus to the regions surrounding these SNPs. Instead, we next expanded our candidate gene search to regions beyond the immediate vicinity of phenotypically-associated SNPs, as described in the next section.

### Candidate gene identification

Regions of CFA 7, 9, 23, and X share synteny with regions of the human genome which harbor loci encoding proteins with functions relevant to DMD/GRMD.

#### CFA7

SNPs were identified in a ~19 Mb region of CFA7, which shares homology with HSA18. In both species, this region comprises a number of genes encoding proteins involved in muscle and/or cardiovascular function. For example, several of these proteins are associated with dystrophin/muscle (dystrobrevin alpha - *DTNA*; laminins alpha and gamma - *LAMA1* and *LAMC1*; lamin a – *LMNA*; and myomesin 1 – *MYOM1*). Others are associated with cardiomyopathy, such as that observed in DMD and GRMD (desmocollin 2 – *DSC2*; desmoglein 2 – *DSG2*). We therefore investigated 11 candidate genes in this section of the dog genome.

### CFA9

A 2 Mb region on CFA9 was associated with TTJ tetanic flexion measurements. This section of CFA9 is homologous to segments of HSA15 and HSA9. Unlike CFA7, this region is not home to many genes with obvious potential roles in GRMD. We investigated one gene, *RAPGEF1*, located on CFA9, which we identified as the most likely to play a role in GRMD of the few genes in this region.

#### CFA23

The significant SNP associations found on CFA23 spotlighted two regions: a ~3 Mb region associated with ECD at 30 stimulations, and a single SNP near the 12.8 Mb position of the chromosome, associated with percent body weight gain from birth to one month of age. Much of CFA23 shares homology with HSA3; we evaluated 3 candidate genes from this chromosome for expression differences in our GRMD cohort.

#### CFAX

We measured transcripts for three different sections of *DMD*, and for additional loci on CFAX based on the locations of associated SNPs. In total, we evaluated expression levels for 7 loci on CFAX.

Genes outside of the regions of initial interest were also considered for validation because of their biological relevance.

#### CFA18

CFA18, homologous to HSA11, was not quite as strongly correlated with GRMD biomarkers – in fact, the 2 SNPs on CFA18 identified in this study were found to be only *nearly* significant (FDR *p*-value = 0.06) and were associated with percent body weight gain from birth to one month of age. However, the chromosome contains several genes encoding proteins with functions pertaining to skeletal muscle phenotypes or neuromuscular junction development; therefore, we investigated the expression levels of 16 genes from CFA18.

#### Other chromosomes

We investigated the transcript levels of 20 more genes from chromosomes that were not found to harbor significantly-associated SNPs. Several of these genes were evaluated as potential “housekeeping” genes for normalization (including *ACTB*, *GUSB*, *PPIA*, and *PSMD2*), and most were not found to be suitable for this role because they showed variable expression levels across dogs. However, we chose to continue to investigate some of these genes as potential candidate modifiers based on their functions in related conditions (for example, *GUSB*; see below). Others of this group of genes encode proteins that have been suggested as modifiers in DMD or *mdx*, and/or are involved in muscle regeneration and degeneration, or were strongly associated in follow-up LMM analyses by GEMMA or SVS (e.g., telomerase reverse transcriptase, *TERT*).

In total, we investigated 58 transcripts for differential expression in GRMD and normal dogs. Gene names, genomic positions and qPCR primer information for these transcripts are listed in Additional file 1: Table S1.

### Correlations between gene expression levels and phenotypic measurements

We used qPCR to measure the expression of candidate “modifier genes” in the cranial tibialis and medial head of the gastrocnemius muscles of the same dogs used for the mixed-model study. Relative expression data were calculated using the ΔΔCt method of Livak and Schmittgen [53], and normalized to the geomean. Of the original set of 58 genes, 12 were dropped from further analysis due to inconsistent measurements across replicates. In total, we measured the expression levels of 46 gene transcripts.

**Fig. 4:**
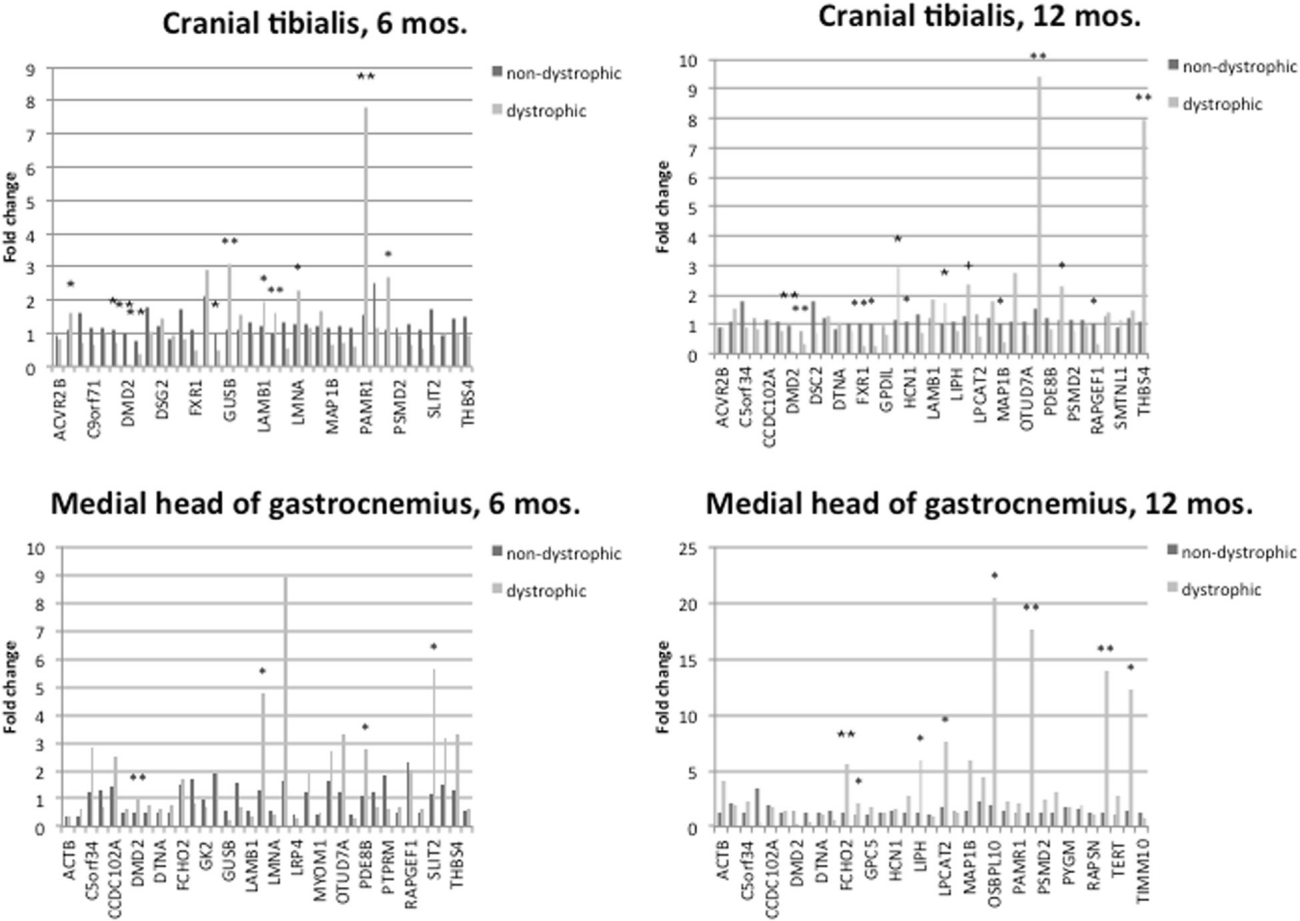
Gene expression levels following normalization against a normal (non-dystrophic) dog and geometric mean [53]. * = significantly different between GRMD and non-dystrophic dogs (unpaired *t* test; *p* < 0.05). ** = very significantly different between GRMD and non-dystrophic dogs (unpaired *t* test; *p* < 0.01). + = near significant (unpaired *t* test; *p* < 0.06)

Fold-change comparisons are shown in Fig. 4 for all genes.

#### Linear regression correlating gene expression with clinical phenotype measurements

**Fig. 5:**
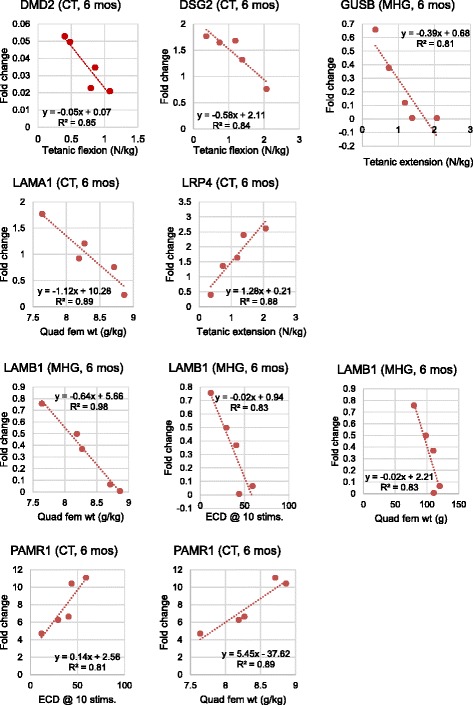
Linear regression analyses performed on the 5 affected 6-month-old GRMD dogs are shown for those genes whose transcript levels (x-axis) correlated with phenotypic measurement values (y-axis). Each plot shows the linear regression equation and correlation coefficient (*R* ^2^). Results are not shown for 12-month-old GRMD dogs because there were only 3 affected dogs in that group. CT = cranial tibialis, MHG = medial head of the gastrocnemius, ECD = eccentric contraction deficiency (at 10 or 30 stimulations), quad fem wt g = quadriceps femoris weight in grams

Linear regression analysis showed that expression levels of some genes correlate directly with biomarker measurement values (Fig. 5). This approach has been used successfully to identify candidate modifiers in other studies in humans and animals [54–58]. These findings increase confidence in the relevance of potential candidate modifier genes. Observations made via linear regression offer novel avenues to explore in future studies of the mechanisms underlying specific GRMD – and DMD – phenotypes.

What is most interesting about the correlations found via linear regression is the specificity of muscle type observed in these relationships: correlations found between transcript levels in the medial head of the gastrocnemius vs those seen in the cranial tibialis. These results reflect the differences in muscle injury in GRMD, further suggesting that future treatment regimens could be tailored to muscle type for maximum effectiveness. Here, all correlations described involve 6-month-old dystrophic dogs (*n* = 5). There were only 3 dystrophic 12-month-old dogs, making linear regression analysis less powerful for this age group; therefore we do not discuss here the analysis for this cohort.

In the cranial tibialis muscle, transcripts from the *DMD2* primer set, located near exons 7–8 of the *DMD* gene, were decreased with greater tetanic flexion values. It is unclear whether any of this transcript was translated into a protein; the transcript was likely subject to nonsense-mediated decay and therefore it is difficult to make a true connection between this biomarker and *DMD2* levels. We found that decreasing levels of desmoglein 2 (*DSG2*) transcript within the cranial tibialis muscle were correlated with increasing measurement values for tetanic flexion. *DSG2* encodes a calcium-binding transmembrane glycoprotein which has been implicated in right ventricular cardiomyopathy [59]. While cardiomyopathy is indeed a part of GRMD disease, here we observed transcriptional activity within skeletal muscle. Next, laminin, alpha 1 (*LAMA1*) transcript levels decreased in the cranial tibialis in tandem with increasing quadriceps femoris weight (when corrected for body weight) at age 6 months. *LAMA1* encodes the laminin a1 chain of the laminin-111 heterotrimer, a protein previously investigated for therapeutic potential in *mdx* mice [60]. Additionally, low density lipoprotein receptor-related protein 4 (*LRP4*) is involved with the maintenance of neuromuscular synapse formation and neuromuscular junction [61]. We associated higher levels of *LRP4* transcript in the cranial tibialis with larger tetanic extension measurements. Finally, transcript levels of a gene called peptidase domain containing associated with muscle regeneration 1 (*PAMR1*) in the cranial tibialis associated linearly with eccentric contraction decrement at 10 stimulations as well as quadriceps femoris weight when corrected for body weight. In both instances, increases in the amount of PAMR1 transcript were correlated with increases in measurement values of these biomarkers. Overall, the cranial tibialis muscle showed the most linearity between transcript levels of candidate modifier genes and biomarker measurement values.

The medial head of the gastrocnemius muscle is affected differently than the cranial tibialis. Not surprisingly, transcript levels of different candidate genes were associated with GRMD biomarkers. In another example of linear correlation, beta glucuronidase (*GUSB*) expression levels in the medial head of the gastrocnemius of 6-month-old GRMD dogs decreased with increased tetanic extension measurements (*R*^2^ = 0.81), which also suggest a relationship between transcript levels of these genes and GRMD phenotype. Also in the medial head of the gastrocnemius of 6-month-old GRMD dogs, transcript levels of laminin, beta 1 (*LAMB1*) correlated nearly perfectly with measurements of quadriceps femoris weight (*R*^2^ = 0.83 for muscle weights themselves, and *R*^2^ = 0.98 when overall body weight was also taken into consideration). In other words, increased weight of the quadriceps femoris was associated with decreased levels of *LAMB1*. Furthermore, *LAMB1* expression correlated well with eccentric contraction decrement measurements after 10 stimulations. *LAMB1* is one of several extracellular matrix glycoproteins and has been previously associated with muscular dystrophies – particularly limb-girdle muscular dystrophy, where *LAMB1* levels have been observed to be decreased [62].

#### Comparisons of expression at different ages

In the cranial tibilalis of 6-month-old dogs, expression was significantly different between GRMD and normal dogs for 11 genes; for 12-month-old dogs, a significant difference was seen for 12 genes (including 6 of the same genes that were differentially expressed at age 6 months). In the medial head of the gastrocnemius, we found 4 genes to have significantly different expression levels between GRMD and normal dogs at age 6 months. 3 of these remained significant in comparisons at 12 months of age, along with 5 additional genes.

#### Comparisons of expression in different muscles and at different ages

While the cranial tibialis and medial head of the gastrocnemius are both located in the distal pelvic limb, their functions differ. The cranial tibialis is a flexor of the tibiotarsal joint, while the medial head of the gastrocnemius is an extensor. Natural history functional data indicate that TTJ flexors are affected relatively early in the disease course and recover to some extent [24]. On the other hand, TTJ extensors are affected later in the disease course. Similarly, the medial head of the gastrocnemius undergoes considerably greater atrophy than the cranial tibialis [20].

The two muscles we evaluated have different (often opposite) functions in GRMD clinical signs, and expression level comparisons between affected and unaffected dogs were found to be significant in *both* muscles for only the *DMD* gene, as expected. DMD2 was significant in both age groups in the cranial tibialis and at 6 months in the medial head of the gastrocnemius, while differential expression of DMD3 was significant at both ages in the cranial tibialis.

Overall, more genes were differentially-expressed in the cranial tibialis than the gastrocnemius. This could be due to the fact that the cranial tibialis is affected earlier in the course of GRMD disease. Some of these differentially-expressed genes had statistically-significant expression differences specifically in one muscle but not the other, an observation which could have implications for the different ways these muscles function in GRMD. Beta glucuronidase (*GUSB*) was originally selected as a possible housekeeping gene for normalization, but surprisingly we found the expression of *GUSB* to vary substantially. We observed that *GUSB* expression levels were increased in the cranial tibialis in GRMD relative to normal dogs at both ages, while levels in the gastrocnemius were fairly equal. Likewise, laminin gamma 1 (*LAMC1*), peptidase domain containing associated with muscle regeneration 1 (*PAMR1*) and peptidylprolyl isomerase A (*PPIA*) expression differences were significant only in the cranial tibialis. However, these differences were significant in both 6- and 12-month-old age groups.

Similarly, 3 genes showed significant differential expression only in the medial head of the gastrocnemius. These included lipase, member H (*LIPH*), phosphodiesterase 8B (*PDE8B*) and slit guidance ligand 2 (*SLIT2*). Each of these genes was found to have significant differences in expression level between dystrophic and non-dystrophic dogs at both ages.

We observed age-specific differential expression for *FXR1* (fragile X mental retardation, autosomal homolog 1) and thrombospondin 4 (*THBS4*). Both of these genes showed the greatest differences in expression level at 12 months of age. This could reflect the increased severity of GRMD disease at 12 months compared to 6 months of age, or it could highlight a specific phase of development during which these genes are most active. The protein encoded by *FXR1* plays a role in myogenesis [63], while *THBS4* is involved in nervous system development.

#### Transcription of the DMD gene

We found measurable transcript levels of the *DMD* gene in the 8 dystrophic GRMD dogs. These levels varied based on the locations of the primers within *DMD*, and muscle type. Paradoxically, in both the cranial tibialis and gastrocnemius, transcript levels were *higher* in GRMD versus non-dystrophic dogs for all three sections of the *DMD* gene. However, in both muscle types, transcript levels were not the same for all three segments of *DMD*.

Statistically-significant up-regulation of *DMD* transcripts was found in the cranial tibialis at age 6 months in dystrophic versus non-dystrophic dogs. By age 12 months, however, only transcripts representing some of the 5’ part of the gene (known here as DMD2, with primers designed across exons 7–8) were found to be significantly increased, both for the cranial tibialis and the medial head of the gastrocnemius. While it is not surprising to find evidence of transcription upstream of the causal GRMD mutation, the findings for primers DMD1 and DMD3 were unexpected. These assays represent sections of the *DMD* gene that are farther downstream (exons 46–47 and 69–70, respectively), yet measurable transcription was observed in the cranial tibialis of 6-month-old dogs. Moreover, these transcripts were significantly up-regulated in GRMD dogs when compared with non-dystrophic dogs.

*DMD* transcripts have been previously identified in the cranial sartorius and vastus lateralis muscles of dystrophic dogs [64] as well as in dystrophic mice [65]; however, these transcripts were more often found to be from the 5’ end of the gene sequence than the 3’end. This imbalance was postulated to explain the observed transcription of *DMD* in these studies, an explanation that may apply to some of our own findings from the cranial tibialis. These and other studies also suggest that these rogue *DMD* transcripts are likely to be subject to nonsense mediated decay and completely nonfunctional at the protein level [65–67]. However, our lab has found evidence of protein expression (M. Schneck, personal communication) which suggests that translation of these DMD transcripts takes place to some extent.

Previous studies have postulated that exon-skipping can occur in human DMD, in cases where a causal point mutation either disrupts enhancer sequences or creates suppressor sequences leading to atypical exon splicing [68, 69]. Such situations could instigate exon skipping, resulting in variable levels of dystrophin production [70–75]. Mutations within certain regions of *DMD* tend to be more prone to this type of phenomena, which may explain some of the phenotypic variation in DMD [69]: alternate start codons or alternate splicing in the 5’ (proximal) end of the *DMD* gene can “rescue” the dystrophin transcript [4, 76–78]. This region includes intron 7, which is highly susceptible to mutation in humans [79, 80]. Importantly, this region is also homologous to that of the GRMD causal mutation [81]. Therefore, it is possible that *DMD* transcription and translation in GRMD dogs can be explained by exon-skipping; we intend to investigate this theory via sequencing and additional protein studies.

## Discussion

The clinical variability seen in DMD suggests that disease outcome is influenced by genetic modifiers of the causal mutation in the *DMD* gene. Genetic modifiers that influence phenotypes can serve as additional druggable targets for treatment. In humans, the search for genetic modifiers is confounded by the large number of mutations associated with DMD and the absence of defined genetic populations. Animal models of the disease are useful for discovery of genetic modifiers because the genetic backgrounds are relatively homogeneous, particularly for the causal *DMD* mutation. However, the use of models that do not resemble the human disease may limit the relevancy of modifiers discovered.

The GRMD colony at Texas A&M University is fairly inbred yet sufficiently genetically diverse to study complex phenotypes such as those observed in DMD. All GRMD dogs are descended from the same founding sire and all share a common causal mutation, but variation is observable in the progression of disease and severity of the phenotype [32]. The breeding strategy employed is such that the Texas A&M GRMD colony possesses many of the advantages of an inbred population (e.g. reduced influence of confounding factors such as environmental variation), but unlike inbred mice, GRMD dogs feature genetic diversity more similar to that found in human populations. The identification of genetic modifiers is therefore simplified by using our GRMD colony and requires a smaller sample size than would a study using humans [82]. Understanding the role(s) of genetic modifiers of GRMD may identify genes and pathways that also modify phenotypes in DMD and reveal novel therapies.

This study represents a preliminary characterization of the GRMD model to identify potential candidate modifier genes. The extensive LD found in many dog breeds allows the efficient identification of causal genetic variants for Mendelian conditions. However, finding modifier genes for more complex phenotypes is less straightforward. Populations where heterozygosity is more prevalent than seen in typical inbred populations add to the challenge, but can provide results that are arguably more relevant to a broader population. The analyses described here are meant to serve as a pilot study for evaluating the likelihood of genetic modifiers of GRMD phenotypes. We anticipate that our findings will provide a starting point for additional studies.

In this paper we have presented evidence for genetic influences on objective phenotypic biomarkers in GRMD. Our study allowed us to compare mildly and severely affected GRMD dogs with high degrees of relatedness without confounding factors such as environmental differences. We used a sample set that included GRMD affected dogs of both genders, but as in previous studies we did not see any significant gender-specific difference in biomarker measurements. With our small cohort of dogs, we identified regions that correlated with GRMD biomarkers in some way, perhaps directly or as a part of a larger pathway. It should be noted that the applicability of the results we describe here are limited to the GRMD model, though the putative candidate genes we have identified provide support for future investigations toward the identification and characterization of modifier genes. Because of the high degree of similarity between GRMD and DMD, such searches are of utmost importance to expand and enhance the relevance and applicability of this animal model for understanding the “minor players” (called “secondary effects” versus “primary” effects of dystrophin deficiency; [83]) of DMD disease variation (e.g., the underlying genetic background affecting variant DMD phenotypes) and for developing novel therapeutics (e.g., trials for drugs and other experimental therapies). While the power of this particular study is limited by the small number of subjects, our results indicate that GRMD phenotypes are, indeed, susceptible to genetic background.

The fact that qPCR values for the same genes differed between muscles suggests that pathways or mechanisms vary for individual muscles. As expected, there was also differential expression with age, reflecting the stage of disease. While this is not surprising, the implications must be considered when evaluating the outcomes of clinical trials. It may be that targeted treatments will be a preferable option for some patients for whom other therapies (e.g. stem cell, gene replacement, or chemical) have failed to produce satisfactory results. In these situations, understanding the involvement of specific sets of genes on different muscles or time points could lead to treatments that, for example, improve one aspect of DMD disease without detrimentally affecting others.

## Conclusions

For monogenic conditions such as DMD that show a spectrum of phenotypes, there is tremendous value in identifying potential modifiers. Such modifiers could influence outcomes of therapeutic trials performed using GRMD as a model for DMD. On the other hand, modifiers may also serve as targets for novel therapeutics. Detecting these genes, therefore, facilitates the role that the GRMD model plays in accelerating DMD treatment development. Characterization of genomic variation underlying GRMD phenotypic variation will present a powerful resource for understanding the molecular causes behind variable DMD phenotypes.

## Abbreviations

CFA, Canis familiaris chromosome; R^2^, correlation coefficient; CT, cranial tibialis; DMD, Duchenne muscular dystrophy; *DMD*, *dystrophin gene*; ECD, eccentric contraction decrement; EMMA, Efficient Mixed-Model Association; EMMA eXpedited, EMMAX; FDR, false-discovery-rate; GeneRIFs, Gene References into Function; GEMMA, Genome-wide efficient mixed-model association; GRMD, Golden Retriever muscular dystrophy; HSA, Homo sapiens chromosome; LMM, linear mixed-model; LD, linkage disequilibrium; MHG, medial head of the gastrocnemius; MAF, minor allele frequency; MDS, multidimensional scaling; quad fem wt g, quadriceps femoris weight in grams; qRT-PCR, quantitative reverse transcriptase PCR; SNP, single nucleotide polymorphism; SVS, SNP and Variation Suite; TTJ, tibiotarsal joint

## Additional files

Additional file 1: Table S1. Primer and assay information for qPCR experiments. The Gene IDs and associated Gene Names are found on the left, followed by their CFA chromosomal locations (chromosome number), and start and end positions (in bp) based on CanFam3.2. The TaqMan assays or SybrGreen primer sequences used for qPCR are presented next, along with the associated UniGene ID (where available). The remaining columns indicate which mRNA and protein sequences are associated with the investigated genes, along with the locations of the qPCR assays themselves and size of the expected amplicon. (XLSX 34 kb) Additional file 2: Table S2. (located at http://dog.genomezoo.net/) SNP associations, listed by chromosomal location, are group by association method (e.g. plink, GEMMA, and SVS). For all associations, phenotypes are given in the leftmost column, followed by information about the SNP/marker and its location, allele frequencies, and statistical information. Additional file 3: Table S3. Variant Effect Predictor output for 90 SNPs found to be significantly associated with phenotypes. The legend for the table is located at the bottom of the table, along with the count numbers for each of the different variants found by Ensembl’s Variant Effect Predictor. Rows for different SNPs and the genes they affect are colored differently, and the font of the gene names are formatted differently, to indicate the muscle types (if any) in which that gene was investigated. (XLSX 35 kb)
